# A Quality Improvement Protocol for Assessing the Quality of Assessments for Children and Adolescents in Crisis

**DOI:** 10.1192/bjo.2025.10356

**Published:** 2025-06-20

**Authors:** Azim Daud, Tauseef Mehdi, Emily Perrin, Mary Curtis-Weight

**Affiliations:** Berkshire Healthcare NHS Foundation Trust, Reading, United Kingdom

## Abstract

**Aims:** Berkshire Healthcare NHS Foundation Trust utilises a Quality Management Improvement System (QMIS) which facilitates a culture of continuous improvement across the Trust. This system includes regular “Huddles” where all staff are encouraged to participate in identifying areas for improvement. Through a Huddle within the Berkshire Child and Adolescent Mental Health Service (CAMHS) Rapid Response Team, concerns were raised about the variable quality of assessments for children and adolescents in crisis. This project was designed to address this concern.

**Methods:** We designed a multifaceted approach to accurately map out the scale of the issue from multiple perspectives to help identify training needs and direct future interventions involving:

1. Designing a quality framework and rating system for reviewing assessments looking at domains agreed by the senior multidisciplinary team (psychiatry, management, psychology and nursing) and informed by existing assessment guidelines. Domains agreed:

Comprehensiveness.

Accuracy and clarity.

Formulation.

Sensitivity and cultural competence.

Document quality.

Rated from 1–5 (1 – poor, 2 – needs improvement, 3 – satisfactory, 4 – good and 5 – excellent).

2. A rating exercise using the framework is to be completed by all assessing clinicians split into two groups (for anonymity), facilitated by senior clinicians. A total of 36 assessments (18 per group) completed in the preceding three months are to be reviewed.

3. Finally, the systemic family therapist would arrange to observe all assessing clinicians in at least one initial assessment to identify and note any other areas for improvement or concern within the assessment itself.

Following the above, information will be collated and analysed to identify specific areas of need within the team’s assessments.

**Results:** We describe a comprehensive approach to review assessment quality within teams, and which encourages the utilisation of multidisciplinary expertise. The framework can be adapted to the needs and multidisciplinary composition of other teams. The crucial aspect is multidisciplinary collaboration – to ensure a holistic assessment of quality.

Involving assessing clinicians in rating assessments is a strength as it allows their perspectives to be included, and simultaneously creates a learning opportunity by attuning them to what is expected of an assessment.

We have also demonstrated the value of having quality improvement systems embedded within the standard work of a team which shares out responsibility and accountability and encourages wider participation.

**Conclusion:** The quality improvement project methodology described can be used by other teams to map out current assessment quality and identify specific target areas for improvement. Further work might include co-production and external validation of our rating guide.

